# NR2C in the Thalamic Reticular Nucleus; Effects of the NR2C Knockout

**DOI:** 10.1371/journal.pone.0041908

**Published:** 2012-07-25

**Authors:** Yuchun Zhang, Andres Buonanno, Robert P. Vertes, Walter B. Hoover, John E. Lisman

**Affiliations:** 1 Department of Biology, Brandeis University, Waltham, Massachusetts, United States of America; 2 Volen Center for Complex Systems, Brandeis University, Waltham, Massachusetts, United States of America; 3 Section on Molecular Neurobiology, National Institutes of Child Health and Human Development, National Institute of Health, Bethesda, Maryland, United States of America; 4 Center for Complex Systems and Brain Sciences, Florida Atlantic University, Boca Raton, Florida, United States of America; Virginia Commonwealth University, United States of America

## Abstract

NMDAR antagonists can evoke delta frequency bursting in the nucleus reticularis of the thalamus (nRT). The mechanism of this oscillation was determined; antagonist blocks an NR2C-like conductance that has low Mg block at resting potential and thus can contribute a resting inward current in response to ambient glutamate. Block of this current hyperpolarizes the cell, deinactivating T-type Ca channels and thus triggering delta frequency bursting. The basis for assuming a NR2C-like conductance was that (1) transcripts for NR2C are abundant in the thalamus and (2) the current-voltage curve of the synaptically evoked NMDAR current has the low rectification characteristic of NR2C. In the current study, we have sought to determine whether the channels that generate the NMDAR current are NR2C-like or are actually comprised of receptors containing NR2C. We studied the current-voltage curve of synaptically evoked NMDAR current in the nRT of NR2C knockout mice. In wild-type mice, the current was weakly voltage dependent, as previously observed in rats. This weak rectification was absent in NR2C KO mice. In contrast, NR2C KO had no effect on the strongly rectifying NMDAR current in pyramidal cells of the prefrontal cortex. These results demonstrate that the low rectification normally observed in the nRT is due to NR2C.

## Introduction

NMDARs are ionotropic glutamate receptors that are comprised of an obligatory NR1 and at least one type of NR2 (2A–2D) or NR3 (3A/3B) subunits [1]. The biophysical properties of NMDAR are largely determined by their subunit compositions. NR2 subunits include NR2A, 2B, 2C, and 2D. These confer different sensitivities to extracellular magnesium and affect the rectification properties of the channel [2]. Specifically, NR2A/2B-containing receptors are highly sensitive to Mg and thus show strong rectification. In contrast, NR2C/2D receptors have weak Mg block and thus low rectification. This low rectification of NR2C has been established in recombinant systems [2] and has been inferred from genetic deletion of NR2A subunit [3]; however, the direct evidence of NR2C has been lacking.

nRT neurons express NR2C transcripts [4], [5]. The synaptically evoked NMDAR current in these cells shows weaker rectification than in cortex and hippocampus [6]. Such weak rectification in nRT neurons is of particular interest because it allows these channels to contribute significantly to resting potential [6] in response to ambient glutamate [7]. Thus, blocking these channels hyperpolarizes the cell; this, in turn, deinactivates T-type Ca channels and results in delta-frequency bursting [6]. These findings have special significance for schizophrenia because delta oscillations in the awake state are elevated [8]. Furthermore, the ability to induce these oscillations by NMDAR antagonist supports the NMDAR hypofunction model of the disease [9], a model supported by the fact that many of the other cognitive and cellular changes observed in the disease can be induced by NMDAR antagonists [10].

The conclusion that delta oscillations are due to an action of NR2C would be important for therapeutic strategies; however, the presence of NR2C in the thalamus and the low rectification characteristic of NR2C cannot be considered definitive evidence for NR2C. Other forms of NR2 are also present in the thalamus [11]. Furthermore, rectification of NR2A/B can be lowered by PKC-mediated channel modulation [12]. To determine whether the NMDARs in the nRT are actually NR2C, we measured NMDAR currents in NR2C knockout mice (Karavanova et al., 2007).

## Materials and Methods

### Brain Slice Preparation

We used a NR2C *nβ*-galactosidase knock-in mouse line in which *nβ*-galactosidase was inserted after the translation initiation site of the Grin2c gene, and the first 11 exons downstream of the initiation methionine were removed by homologous recombination to render the NR2C gene inactive (Karavanova et al., 2007). Mice were housed under a 12-h light/dark cycle in a temperature- and humidity-controlled environment with free access to food and water. The animals (∼3 weeks old) were sacrificed under fluothane anesthesia. The brains were rapidly removed and were cut into 300–350 µm thick horizontal slices with a vibratome (Leica VT 1000S, Nussloch, Germany) in an oxygenated ice-cold solution containing (in mM): NaCl 124, KCl 2.5, NaHCO_3_ 26, NaH_2_PO_4_ 1.25, Dextrose 10, CaCl_2_ 2.5, and MgSO_4_ 4. Slices containing the nRT were collected and incubated for at least 1 h before transferring into the chamber for recording. Experimental protocols were approved by the institutional animal care and use committees at the Brandeis University.

**Table 1:** Deactivation time-constant of the NMDAR-mediated synaptic response.

	Wild type	NR2C knockout
**PFC**	225.3±28.2 ms	219.1±29.5 ms
**nRT**	256.4±37.7 ms	231.0±36.6 ms
**Reuniens**	240.7±33.3 ms	

## Discussion

### Electrophysiology

Patch electrodes had resistances of 3–5 MΩ. The internal solution contained (in mM): CsCl 43, CsMeSO_4_ 92, TEA 5, EGTA 2, MgCl_2_ 1, HEPES 10, and ATP 4. pH was adjusted to 7.2–7.4 with CsOH, and the final osmolarity was ∼290 mOsm. Brain slices were immersed in oxygenated artificial cerebrospinal fluid with a flow rate of 2–3 ml/min. The nRT was visually identified using dark field illumination and a CCD camera. Voltage-clamp recordings were performed with an Axopatch 200B amplifier (Molecular Devices, Foster City, CA, USA). Signals were digitized at a sampling rate of 5 kHz and were filtered at 2 kHz using a data acquisition program (Igor Pro 5.0, Wavemetrics, Oregon, USA).

The *I-V* curves of the NMDAR-EPSCs were fit by the equation: I  =  a*g*(V−r)/(a+[Mg^2+^]_o_*exp(-V*f), in which V is holding potential, r is reversal potential, g is the conductance at +40 mV, a is the Mg dissociation constant in the absence of transmembrane voltage, and f represents the product of the fraction of membrane voltage at the blocking site times the constant ZF/RT. For wild-type PFC neurons, a  = 4.0645±0.639; g  = 0.027404±0.000673; r  = 3.3662±0.441; f  = 0.66673±0.00335. For wild-type nRT neurons, a  = 6.0846±2.99; g  = 0.024772±0.002; r  = 1.2585±1.36; f  = 0.046933±0.00681. For wild-type reuniens neurons, a  = 3.5344±0.6886; g  = 0.027451±0.004; r  =  −1.2476±0.488; f  = 0.0545±0.0032. For NR2C-KO PFC neurons, a  = 3.5816±1.42; g  = 0.027921±0.002; r  = 5.135±1.17; f  = 0.061373±0.00774. For NR2C-KO nRT neurons, a  = 2.6801±0.372; g  = 0.02976±0.00089; r  = 4.1866±0.436; f  = 0.0527±0.0024. The decay of NMDAR-EPSC was fit by the sum of two exponential functions: *A*(*t*)  =  *A*_slow_exp(−*t*/τ_slow_) + *A*_fast_exp(−*t*/τ_fast_), in which τ_slow_ and τ_fast_ are the decay time constants of the slow and fast component and *A*_slow_ and *A*_fast_ are their respective amplitudes. The decay time constants were averaged using τ_slow_[*A*_slow_/(*A*_slow_ + *A*_fast_)] + τ_fast_[*A*_fast_/(*A*_slow_ + *A*_fast_)].

The *I-V* curves (10 mV increments) of the NMDAR-EPSCs were fit by the equation: *I*  =  [*ag*_max_(*V*−*V*_r_)]/[*a* + [Mg^2+^]_o_exp(−*V*δZF/RT)], in which *V* is the holding potential, *V*_r_ is the reversal potential, *g*_max_ is the conductance at +40 mV, *a* is the Mg dissociation constant in the absence of transmembrane voltage, and δ represents the fraction of membrane voltage at the blocking site.

### Statistics

The data are presented as mean ± standard error. The two-tailed paired t test was used for two-group comparisons. ANOVA followed by Tukey’s test was used for multigroup comparisons. The difference was considered significant when P<0.05.

## Results

**Figure 1:**
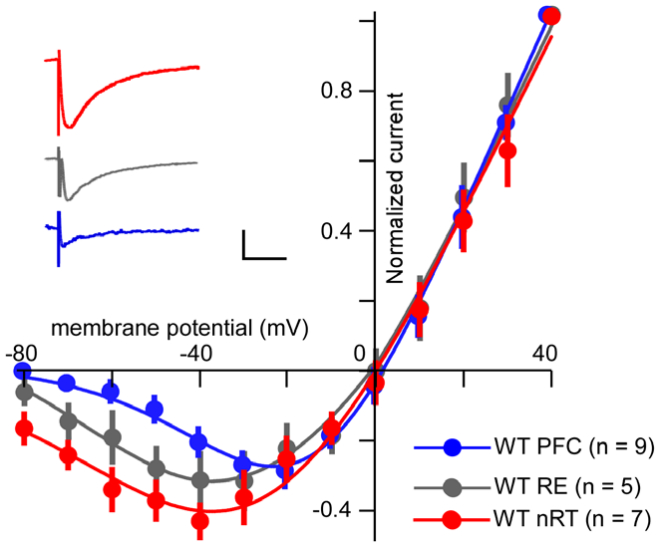
The current-voltage relationships of the synaptically induced NMDAR-mediated response recorded from slices of wild-type PFC, nRT, and RE neurons. Insets show representative traces at holding potential of −60 mV; scale bars: 200 ms, 50 pA.

We evoked NMDAR-mediated EPSCs in the presence of CNQX (25 µM) and bicuculline (20 µM) by stimulating the internal capsule, which primarily activates glutamatergic corticofugal fibers projecting to the nRT. The holding potential was changed step-wise from −80 to +40 mV. To quantify the voltage dependence of NMDAR currents, we normalized the currents at various voltages to that at +40 mV. As shown in Figure 1, WT nRT neurons displayed weak rectification at negative membrane potentials, confirming similar results in rats [6]. Fitting I-V relations indicated that the inward NMDAR current had a peak at −37.3 mV and declined as membrane voltage became more negative; this aspect of rectification is quantified by the “relative current,” which compares that at −60 mV to that at +40 mV; the relative current was −0.34±0.06; n = 7. In contrast, in the PFC (Fig. 1), the peak current was at −22.1 mV, and the rectification current was stronger (relative current  = −0.07±0.02, n  = 7, *P*<0.01;).

**Figure 2:**
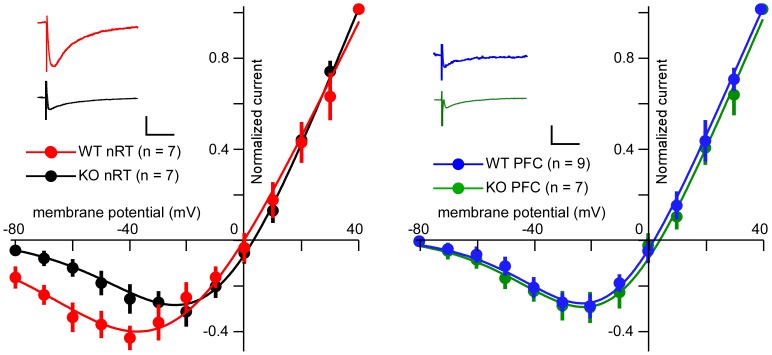
The current-voltage relationships of the synaptically induced NMDAR-mediated response in the nRT (left panel) and the PFC (right panel) recorded from slices of WT and NR2C knockout mice. Insets show representative traces at holding potential of −60 mV; scale bars: 200 ms, 50 pA.

We then made similar measurements in NR2C knockout mice. First, we found that NR2C deletion had little impact on the shape of I-V curves recorded from PFC pyramidal neurons; the peak negative currents were at −22.5 mV, and the relative currents were −0.09±0.02 ) (Fig. 2, right). However, the I-V curves of nRT neurons were dramatically changed in slices from NR2C knockout mice (Fig. 2, left); the relative current was −0.12±0.03, which is significantly different from the WT. The peak negative current occurred at −24.2 mV. Because the voltage of the stimulus electrode is set to evoke a standard current, no information is available about the absolute magnitude of the NMDAR-mediated response in the KO relative to the WT.

Although NR2C transcripts, as seen in the Allen Brain Atlas, are very abundant in thalamus, the expression is not uniform. Notably, the nucleus reuniens, a midline thalamic nucleus of special interest because of its innervations of the hippocampus [13], shows expression much lower than the nRT. Consistent with this, the synaptically evoked NMDA current in cells of this nucleus had stronger rectification than in the nRT but weaker rectification than in cortex (Fig. 1). The relative current at −60 mV is 0.19±0.08 in reuniens neurons, which is significantly different from both nRT and PFC neurons (Fig. 1). The peak negative current in the reuniens occurred at −38.6 mV.

In addition to the rectification property, we also measured the deactivation time-constant of NMDAR current (see Materials and Methods). As shown in Table 1, NR2C knockout did not significantly change the deactivation time-constant of either nRT or PFC neurons.

Our results show that knockout of NR2C eliminates the low rectification of the synaptically evoked NMDAR currents normally observed in cells of the nRT. This effect of the knockout, together with the evidence that nRT neurons actually contain NR2C RNA [4], [14], leads to the definitive conclusion that NMDAR currents in these cells are mediated by NR2C.

The most distinct properties of NR2C, which has been most extensively studied in the cerebellar neurons, is the lower sensitivity to Mg^2+^, which results in reduced rectification [15], [16]. NR2C is expressed in very few regions in neurons, specifically in the thalamus and in granule cells of the cerebellum [4], [5]. However, it is of interest that even within the thalamus there is considerable heterogeneity of NR2C RNA. One region with relatively low RNA levels is the nucleus reuniens [4], [14]. Consistent with the low expression, the rectification in these cells was higher than in the nRT (although still lower than in PFC).

Our results strengthen the case for considering NR2C a potential therapeutic target for treating schizophrenia. Because NR2C expression is present in relatively few brain areas, it is an attractive target for pharmacological treatment. Given the evidence for NMDAR hypofunction in schizophrenia [9], [10], [17], NR2C agonist are of particular interest. It has been found that a class of novel tetrahydroisoquinolines can specifically increase NR2C/2D opening frequency without affecting NR2A/B. In the presence of both glutamate and glycine, the maximal potentiation effect for NR2C is about 97% [18]. Thus, this class of compounds has the potential for therapeutic action for schizophrenia.
